# Integrating micro-needle jet injection and sustained GLP-1 therapy with structured feeding: a comprehensive strategy for obesity management

**DOI:** 10.1080/10717544.2025.2557938

**Published:** 2025-09-09

**Authors:** Chen Zhang, Luoxin Long, Hong Hu, Xinjin Zhou, Lindsey F. Mao, Jing Wang, Aoran Zhang, Yuji Wang, Yi Yan, Shanhong Mao

**Affiliations:** a School of Pharmaceutical Sciences, Capital Medical University, Beijing, China; b School of Light Industry Science and Engineering, Beijing Technology and Business University, Beijing, China; c School of Biomedical Engineering, Capital Medical University, Beijing, China; d Department of Pathology, Baylor University Medical Center at Dallas, Dallas, TX, USA; e Nomedel USA, LLC, Dublin, CA, USA; f School of Food and Health, Beijing Technology and Business University, Beijing, China

**Keywords:** GLP-1 receptor agonists, micro-needle jet injection (MNJI), needle-free jet injection (NFJI), initial dispersion rate (IDR), sustained release

## Abstract

Obesity is a global health crisis strongly linked to increased risk of type 2 diabetes, cardiovascular diseases, and other metabolic disorders. Glucagon-like peptide-1 (GLP-1) has emerged as an effective macromolecular therapeutic agent for weight management. This study addressed obesity management from three distinct perspectives: enhancing drug dispersion and bioavailability through a novel drug delivery device, extending drug half-life by developing sustained-release formulations, and sustaining the weight loss through implementation of structured dietary protocols. A new technology, micro-needle jet injection (MNJI) was developed to deliver both standard semaglutide formulations and highly viscous sustained-release formulations, achieving 100% subcutaneous delivery with predictable results. Modulation of MNJI parameters enabled the generation of various dispersion profiles, resulting in higher bioavailability compared to both needle injection (NI) and needle-free jet injection (NFJI). Sustained-release formulations, effectively administered via MNJI, exhibited higher bioavailability than the non-sustained release formulation, and positively impacted weight management efficacy in two distinct ways. First, a single injection achieved the same weight loss as five daily administrations of non-sustained release formulation. Second, a subsequent injection of the sustained-release formulations resulted in a further body weight reduction to 18%, contrasting sharply with the plateau at 13% observed in the standard formulation administered daily (*p* < 0.05). Finally, dietary management, particularly time-restricted feeding, successfully maintained weight loss at ∼18% below baseline levels. Collectively, the combination of MNJI delivered sustained-release formulations and structured dietary protocols offers a promising and patient-friendly strategy for long-term obesity management, improving both adherence and therapeutic outcomes.

## GRAPHICAL ABSTRACT

**Figure:**
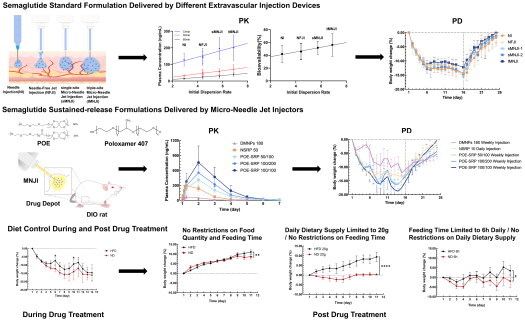

## 1. Introduction

Obesity is an escalating global health crisis that places a substantial burden on healthcare systems worldwide (Smith and Smith 2016). Excessive consumption of high-fat and calorie-dense foods contributes to body fat accumulation (Duan et al. 2018), which in turn increases the risk of numerous clinical complications, including type 2 diabetes (T2D), cardiovascular disease, hypertension, dyslipidemia, and nonalcoholic steatohepatitis (Hinnen 2018; Chang et al. 2019; Newsome et al. 2021; Valenzuela et al. 2023; Rajagopal and McGill 2024). Effective obesity management requires sustained adherence to scientifically formulated dietary plans, significant caloric restrictions, and rigorous, targeted exercise regimens (Li et al. 2022). However, maintaining such lifestyle changes is exceptionally challenging for most individuals, making pharmacological intervention a more practical and widely adopted approach (Grunvald et al. 2022).

Glucagon-like peptide-1 (GLP-1) has recently emerged as an effective macromolecular drug therapeutic, achieving an average weight loss of up to 15% while simultaneously reducing the risk of cardiovascular events (Wharton et al. 2023; Lingvay et al. 2024). Several GLP-1 analogs, such as semaglutide, liraglutide, exenatide, and lixisenatide have been commercialized (Gotfredsen et al. 2014; LaRue and Malloy 2015; Tomlinson et al. 2016; Dhillon 2018; Jones et al. 2019; Oh et al. 2023). Both preclinical and clinical data indicate that GLP-1 facilitates weight loss through multiple mechanisms, including direct activation of proopiomelanocortin/cocaine and amphetamine-regulated transcript (POMC/CART) neurons, and indirect inhibition of neuropeptide Y/agouti-related peptide (NPY/AgRP) neurons (Ghidewon et al. 2022).

Natural GLP-1 is rapidly degraded in circulation by dipeptidyl peptidase IV (DPP-4), with a half-life of only 1–2 min (Chuong et al. 2023). Consequently, frequent injections are required to maintain therapeutic plasma drug concentrations, often leading to poor patient compliance (He et al. 2017). Common strategies to prolong the plasma half-life of GLP-1 include chemical modifications and sustained-release formulations (Schneider et al. 2017; Gallo et al. 2022). Among GLP-1 receptor agonists, semaglutide exemplifies the success of chemical modifications. Semaglutide uses α-aminoisobutyric acid (Aib) to replace alanine (Ala) at position 8 on the GLP-1 peptide chain, masking the hydrolysis site of DPP-4, and preventing rapid degradation by the peptidases (Knudsen and Lau 2019). At lysine 26, semaglutide is derived with a hydrophilic spacer and a C18 fatty di-acid, enabling non-covalent binding to plasma albumin, thereby increasing molecular weight and reducing renal clearance (Gallo et al. 2022). In addition, lysine (Lys) is replaced with arginine (Arg) at position 34 to enhance side chain stability (Lau et al. 2015). These chemical modifications have extended the half-life in humans from 1 to 2 min to 165–183 h (Dhillon 2018).

Sustained-release of GLP-1 involves forming a drug reservoir under the skin by encapsulating the drug within various carrier materials (Agarwal and Rupenthal 2013). The slow dissolution, diffusion, penetration, ion exchange, or degradation of the carrier materials promote gradual releases of the drug to achieve a continuous supply of drug over time (Varanko et al. 2020). Recently, Appel et al. developed nanoparticle (NP) hydrogel as a drug delivery carrier by *in situ* combining dodecyl-modified hydroxypropyl methyl cellulose (HPMC-C12) with biodegradable nanoparticles composed of PEG-PLA (d’Aquino et al. 2023). After loading semaglutide into the NP hydrogel, the slow dissolution of the hydrogel at the injection site allows a continuous release of the drug in rats (d’Aquino et al. 2023).

Despite the success of sustained-release formulations in various clinical applications, their use in GLP-1 has been hindered by the controllability and practicality of drug delivery. Needle injection (NI) has been used to deliver the commercial standard GLP-1 formulations (Hirsch et al. 2010; LaRue and Malloy 2015), but it is ineffective for highly viscous, semi-solid sustained-release formulations (Watt et al. 2019). To overcome this limitation, we developed a novel micro-needle jet injection (MNJI) technology that enables delivery of highly viscous materials with precise targeting (Long et al. 2025).

In this study, a standard GLP-1 formulation was injected subcutaneously using 3 different delivery technologies: needle injection (NI), needle-free jet injection (NFJI), and micro-needle jet injection (MNJI). The effects of these delivery technologies on delivery efficiency, safety, pharmacokinetics and pharmacodynamics were evaluated using a diet-induced obesity (DIO) rat model. Subsequently, two types of sustained-release formulations, an erodible polymer system based on poly (ortho esters) (POE) and a thermal-sensitive gelation (TSG) system based on poloxamer 407, were developed. The safety, pharmacokinetics, and pharmacodynamics of these unique formulations, as well as the MNJI-based drug-device combinations, were investigated using a DIO rat model, with comparison made to the NI-based standard GLP-1 formulation and the dissolving microneedle patches (DMNPs). Lastly, the impact of diet control on the effectiveness of weight loss was systematically studied during and after the drug administration. A combined protocol of sustained-release formulation and diet control was proposed as an effective and long-term obesity management.

## 2. Materials and methods

### 2.1. Standard formulation

The semaglutide active pharmaceutical ingredient (API; C005A-D230301, 99% pure) used in this study was obtained from Hubei Jianxiang Biopharmaceutical Co., Ltd. A blank diluent consisted of 1.42 mg/mL of disodium hydrogen phosphate dihydrate (C16390483, Shandong Keyuan Biochemical Co., Ltd.) and 8.25 mg/mL of sodium chloride (C17087130, Shanghai Macklin Biochemical Co., Ltd.) was prepared using injection-grade water. The pH of the blank diluent was adjusted to 7.4 using either 0.5 mol/L hydrochloric acid or 1 mol/L sodium hydroxide solution. The final formulation was prepared by accurately weighing semaglutide and dissolving it in the blank diluent to a single dose of 10 nmol/kg per rat with a concentration of 0.025 mg/0.3 mL.

### 2.2. Sustained-release formulations

In this experiment, two different types of sustained-release formulations were prepared. The detailed description of the standard formulation and the sustained-release formulations are provided in Tables 1 and 2.

**Table 1.** PK and PD studies of standard formulation.

	Group	Material	Dosage
1	ND	Saline, 300 μL	1 injection (PK), 15 daily injections (PD)
2	HFD	Saline, 300 μL	1 injection (PK), 15 daily injections (PD)
3	NI	Standard formulation, 300 μL	10 nmol/kg, 1 injection (PK), 15 daily injections (PD)
4	NFJI	Standard formulation, 300 μL	10 nmol/kg, 1 injection (PK), 15 daily injections (PD)
5	sMNJI-1	Standard formulation, 300 μL	10 nmol/kg, 1 injection (PK), 15 daily injections (PD)
6	sMNJI-2	Standard formulation, 300 μL	10 nmol/kg, 1 injection (PK), 15 daily injections (PD)
7	tMNJI	Standard formulation, 300 μL	10 nmol/kg, 1 injection (PK), 15 daily injections (PD)

sMNJI-1: single micro-needle jet injection with small injection nozzle; sMNJI-2: single micro-needle jet injection with large injection nozzle; tMNJI: three-sites micro-needle jet injection.

**Table 2.** PK and PD studies of sustained-release formulations.

	Group	Material	Dosage
1	ND	Saline, 300 μL	15 daily injections
2	HFD	Saline, 300 μL	15 daily injections
3	NSRP 10	Standard formulation, 300 μL	10 nmol/kg, 1 injection (PK), 15 daily injections (PD)
4	POE-SRP 50/100	Sustained-release formulation, 100 μL	50 nmol/kg, 1 injection (PK), 1 injection in every 5 days (PD)
5	POE-SRP 100/200	Sustained-release formulation, 200 μL	100 nmol/kg, 1 injection (PK), 1 injection in every 5 days (PD)
6	POE-SRP 100/100	Sustained-release formulation, 100 μL	100 nmol/kg, 1 injection (PK), 1 injection in every 5 days (PD)
7	DMNPs	Sustained-release formulation	180 nmol/kg, 1 application (PK), 1 application in every 5 days (PD)

*Note:* ‘NSRP’ refers to non-sustained-release preparations (i.e. conventional injectable formulations). The number ‘10’ indicates a dosing level of 10 nmol/kg/day. The notation ‘50/100’ means a single-dose administration of 50 nmol/kg in a volume of 100 μL.

#### 2.2.1. Poly (ortho ester)-based formulation

**Figure 1.**
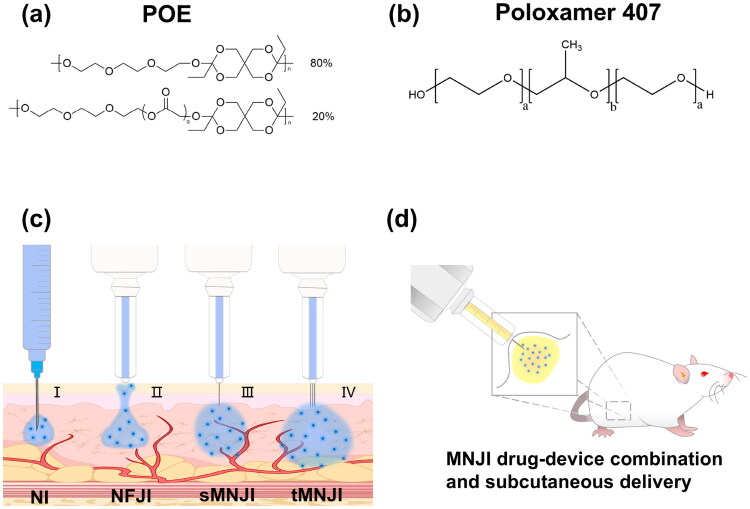
(a) Chemical structure of poly (ortho ester). (b) Structural formula of poloxamer 407. (c) Schematic diagram of different drug delivery technologies: needle injection (NI), needle-free jet injection (NFJI), micro-needle jet injection (MNJI), and three-site micro-needle jet injection (tMNJI). (d) MNJI device preloaded with sustained-release formulation for subcutaneous injection into a DIO rat.

The first sustained-release formulation utilized poly (ortho esters) (POE, AP-20240313-D2, Beijing Nuokangda Pharmaceutical Technology Co., Ltd.) as a carrier. POE is a biodegradable polymer that is highly suitable as an erodible delivery carrier for extending drug efficacy. In a biological environment, POE undergoes gradual hydrolysis at the polymer-water interface, resulting in sustained release of the encapsulated drug (Einmahl et al. 2000). This type of erodible drug carrier can function as an implantable device that does not require removal once the drug has been released (Hatefi and Amsden 2002). The POE used in this study consisted of alternating residues derived from the reaction of diene acetal and diol, with each diene acetal-derived residue separated from the adjacent diol residue (Figure 1(a)). POE is a highly hydrophobic polymer, while semaglutide is a GLP-1 analogue modified with hydrophobic fatty acids. During the preparation of polymer delivery carrier, the fatty acid moieties in semaglutide can non-covalently bind to the hydrophobic polymer chain in POE to suppress the diffusion of drug from delivery carrier (Einmahl et al. 2001).

Table 2 describes the POE sustained-release formulations used in this study. A mixture of 65% POE and 35% triacetin (CTP388, Shanghai Bide Pharmaceutical Technology Co., Ltd.) was used as the sustained-release polymer carrier. Rheological analysis using a rotational rheometer (NETZSCH-Gerätebau GmbH) revealed an optimized viscosity of ∼1809 MPa·s and a density of ∼1.2488 g/mL (Figure 1(b)). The POE sustained-release formulations were made by loading semaglutide at the desired dose into the polymer carrier, stirring at 70 °C for thorough mixing until homogeneous solutions were obtained.

#### 2.2.2. Thermosensitive gel (TSG)-based formulation

The second sustained-release formulation used a thermosensitive gel of poloxamer 407 (M211101, Shanghai Tairan Biotechnology Co., Ltd.) as a carrier for sustained-release preparations. Poloxamer 407 is a family of triblock copolymer with a center block of hydrophobic polypropylene oxide (PPO), flanked by two hydrophilic polyethylene-oxide (PEO) blocks (Kweon et al. 2003). Poloxamer 407 exhibits a unique thermal-reversible gelation behavior. As the temperature increases, the hydrophobic PPO repeat units dehydrate, causing the poloxamer 407 copolymer chains to aggregate into a micelle structure via hydrophobic interactions, thereby triggering the solution-to-gel phase transition (Fakhari et al. 2017). In this study, the specially formulated Poloxamer 407 system exists in a sol state at 0–8 °C (with a viscosity of ∼66 mPa·s) and transitions to a gel state at ∼25 °C (with a viscosity of ∼3138 mPa·s). This thermal-reversible gelation property makes poloxamer 407 an ideal candidate of carrier materials for delivering small molecules, peptides, and biomolecules (Ricci et al. 2005). The safety of the Poloxamer 407 system has been tested by Amiji et al., who reported a survival rate exceeding 91% in tumor-bearing animals 15 days post-administration of a paclitaxel-poloxamer 407 gel formulation (Amiji et al. 2002). In the present study, semaglutide was incorporated at low temperature into the poloxamer 407 sol and stored in prefilled ampoules to form the semaglutide sustained-release formulations. High-speed injection of the MNJI technology allows a low temperature operation to ensure the TSG formulations remain in sol state during the delivery for optimal injectability.

### 2.3. Drug delivery technologies

Three different extravascular drug delivery technologies were employed in this study, including traditional needle injection (NI, Guangxi Beilunhe Medical Industry Group Co., Ltd.), needle-free jet injection (NFJI, QS-P, Beijing Quinovare Medical Technology Co., Ltd.), and micro-needle jet injection (MNJI, prototype from Mao’s Lab). MNJI combines the high driving force of NFJI and the skin penetration ability of NI (Long et al. 2025), offering a promising approach for the extravascular delivery of high viscosity drugs. All delivery devices used in this study were designed for subcutaneous injection. The administration site was set on both sides of the rat abdominal region, and injections were alternated between the left and right sides throughout the study. This approach was intended to prevent repeated administration at the same site, which may lead to lipohypertrophy or induration. Previous research has demonstrated the impact of dispersion at delivery on immunogenicity of COVID-19 vaccines (Mao et al. 2023) and on biologic drugs (Sequeira et al. 2019). Since different initial dispersion rates (IDR) are unique outcomes of the above-mentioned delivery technologies (Mohizin and Kim 2018), three MNJI configurations, in addition to NI and NFJI, were employed to generate a different range of IDRs at delivery site. The delivery devices and configurations are listed in Table 3.

**Table 3.** Different injection parameters of the delivery devices.

Devices	Description	Inner diameter	Driving pressure
NI	Needle injector	232 μm	∼0 MPa
NFJI	Needle-free jet injector	150 μm	14 MPa
sMNJI-1	Single-site micro-needle jet injector	133 μm	20 MPa
sMNJI-2	Single-site micro-needle jet injector	232 μm	20 MPa
tMNJI	Three-site micro-needle jet injector	105 μm for each needle	23 MPa

*Note:* The ‘similar driving pressure’ refers to the fact that the injection devices apply the same magnitude of driving force.

### 2.4. Drug delivery efficiency

Before pharmacokinetic and pharmacodynamic studies, both the standard and highly viscous sustained-release formulations underwent drug delivery efficiency testing. The effect of hair interference on delivery efficiency was also investigated using the standard semaglutide formulation. In this study, delivery efficiency (*DE*) was defined as the proportion of the drug successfully injected into the body (Equation 1). Here, *M_dose_* represented the total mass of the liquid, while *M_injected_* represents the mass of the liquid successfully injected into the body.

(1) $$\text{DE}=\frac{M_{\text{Injected}}}{M_{\text{dose}}}\times 100\%$$

### 2.5. Initial dispersion rate

**Figure 2.**
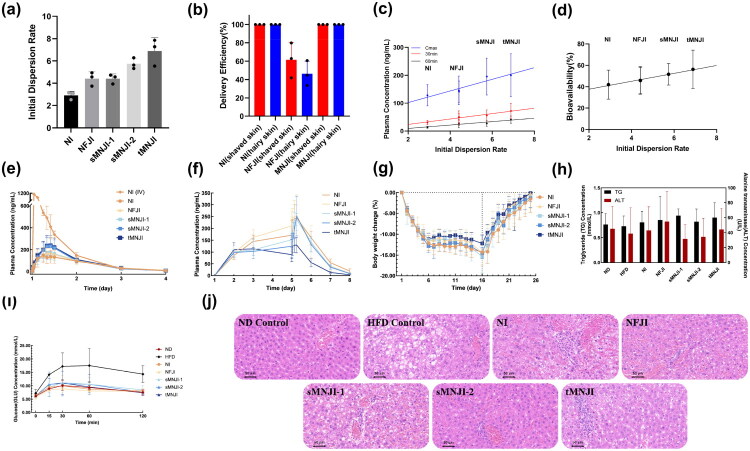
PK and PD results of the standard formulation. (a) Initial dispersion rate (IDR) of different delivery technologies. (b) Delivery efficiency of needle injection (NI), needle-free jet injection (NFJI), and micro-needle jet injection (MNJI) devices. The injection parameters for NFJI are a nozzle inner diameter of 150 μm and an injection pressure of 14 MPa. The injection parameters for MNJI include a needle inner diameter of 232 μm, an injection pressure of 14 MPa, and a micro-needle length of 6 mm. (c) Relationship between IDR and *C*_max_, 30-min plasma concentration, and 60-min concentration. (d) Correlation between IDR and bioavailability. (e) Plasma concentration after single dose subcutaneous injection. (f) Plasma concentration after multi dose subcutaneous injections. (g) Body weight change curve. (h) Blood biochemistry of triglycerides (TG). (i) Changes of blood glucose in rats during glucose tolerance test (GTT). (j) Hematoxylin-Eosin (HE) staining of liver.

Since dispersion at delivery site has impact on drug efficacy, a quantitative metric, Initial Dispersion Rate (*IDR*), was introduced as a metric for dispersion (Equation 2). IDR is defined as the ratio of the dispersed volume of the injected liquid (*V_dispersion_*) to its original volume (*V_original_*). *V_dispersion_* was measured by a nanosponge-gel model described in our previous work (Mao et al., under review). In the present study, IDRs were measured at a consistent dose of 0.30 mL standard semaglutide formulation, on 5 different devices shown in Figure 2(a). These IDR values will be used to explain the pharmacokinetic (PK) and pharmacodynamic (PD) differences of standard semaglutide formulation delivered by these devices.

(2) $$\text{IDR}=\frac{V_{\text{dispersion}}}{V_{\text{original}}}$$

### 2.6. Diet induced obesity animal model

Diet Induced Obesity (DIO) refers to an obese rat model induced by a high-fat diet. Excessive intake of fatty acids increases fat synthesis in the body, leading to substantial body fat accumulation and subsequent obesity. Male Sprague-Dawley rats (Beijing Huafukang Biotechnology Co., Ltd.) were used to establish the model. Following a one-week of adaptive feeding, the male SD rats were randomly divided into a normal diet group (D12492, Beijing Huafukang Biotechnology Co., Ltd.) and a high-fat diet group (D12450B, Beijing Huafukang Biotechnology Co., Ltd.). The high-fat diet group was maintained on the high-fat diet for 20 weeks to induce obesity. Successful DIO induction was confirmed when the average body weight of the high-fat diet group exceeded that of the normal diet group by 20%.

### 2.7. Animal grouping and dosing protocols

Bioavailability was evaluated using intravascular needle injection (IV) as baseline (Table 4). DIO rats were randomly assigned to the IV group and the subcutaneous group for pharmacokinetics study using the semaglutide standard formulation. Pharmacokinetics and pharmacodynamics studies of non-sustained-release formulations were conducted using the semaglutide standard formulation and different delivery technologies (Table 1). DIO rats were randomly assigned to the following groups: high-fat-diet (HFD) and normal-diet (ND) control groups, a needle injection group (NI), a needle-free jet injection group (NFJI), and three micro-needle jet injection groups (sMNJI-1, sMNJI-2, and tMNJI, Table 1). All experimental groups, except HFD and ND, were administrated daily with a single dose of 10 nmol/kg semaglutide formulation. Rats in ND and HFD groups received subcutaneous injections of normal saline (Biosharp^®^, Beijing Lanjieke Technology Co., Ltd.). During the study, all rats were maintained on a high-fat diet, except for the ND group, which was fed with normal diet. Materials were injected subcutaneously in the shaved lower back region of the rats. The pharmacokinetics and pharmacodynamics study design of sustained-release formulations is detailed in Table 2. DIO rats were randomly divided into 7 groups. The normal diet (ND), high-fat diet (HFD), and standard formulation (NSRP 10) control groups received 15 daily injections of saline and semaglutide standard formulation, respectively. POE-SRP groups received 1 injection of the 65%POE/35%triacetine sustained-release formulation every 5 days, for a total of 3 injections. The DMNP group received one application of the micro-needle patch with semaglutide loading of 180 nmol/kg every 5 days, for a total of 3 applications. The experiment was conducted for a total of 15 days, during which the general conditions of the rats were monitored and recorded daily. At the end of the experiment, the rats were fasted overnight and their body weight, waist circumference, and body length were measured. The collected data were then used to calculate the Lee index (Equation 3).

(3) $$\text{Lee}\;\prime \;s\;\text{index}=\frac{\sqrt[{3}]{\text{Body}\;\text{weight}(g)}}{\text{Body}\;\text{length}(\text{cm})}\times 10^{3}$$

**Table 4.** Bioavailability study.

	Group	Material	Dosage
1	IV	Standard formulation, 300 μL	10 nmol/kg, one intravascular injection
2	Subcutaneous	Standard formulation, 300 μL	10 nmol/kg, one subcutaneous injection

*Note:* The injection method used in the experiment was traditional needle injection.

### 2.8. Pharmacokinetic (PK) studies

The concentration of semaglutide in rat plasma was determined using a Waters Xevo^®^ TQ-XS triple quadrupole liquid chromatography-tandem mass spectrometry system. The analysis was performed on an ACQUITY UPLC^®^ BEH C18 column (2.1 × 50 mm, 1.7 μm) maintained at 40 °C. The mobile phase consisted of (A) 0.1% formic acid in water and (B) 0.1% formic acid in acetonitrile, delivered at a constant flow rate of 0.5 mL/min with a 10 μL injection volume. A gradient elution program was employed as follows: 70% A (0.00–0.20 min), 70–35% A (0.20–1.60 min), 35–5% A (1.60–1.65 min), 5% A (1.65–2.00 min), 5–70% A (2.00–2.01 min), and 70% A (2.01–2.50 min). Mass spectrometric detection was conducted in positive electrospray ionization mode (ESI+), monitoring the specific transition of m/z 1029.3 → 1238.2 for semaglutide quantification. The total run time was 2.5 min per sample. All solvents and reagents used were of LC-MS grade.

The pharmacokinetic studies employed a grouping strategy outlined in Section 2.7, with intravascular needle injection serving as the baseline for bioavailability calculation. A single dose of either the semaglutide standard formulation or the sustained-release formulation as described in Sections 2.1 and 2.2 was used to measure PK results and calculate bioavailability. Blood samples were collected before administration and at 0.5, 1, 3, 6, 8, 10, 12, 24, 48, 72, 96, 120, and 144 h post-administration. Plasma concentration was also collected in the multiple administration groups, with daily semaglutide administration for five consecutive days, while blood samples were collected before administration and on days 3 and 5 both before the injections, as well as at 0.5, 1, 3, 6, 24, 48, and 72 h post-administration.

Whole blood samples (0.2–0.3 mL) were drawn from the jugular vein each time and placed in an anticoagulant tube containing EDTA-K2. After centrifugation at 4 °C at 3000 rpm for 15 min, the plasma was harvested and stored at −80 °C for subsequent analysis. Bioavailability was determined by dividing the amount of drug reaching systemic circulation in each subject by the amount of drug reaching systemic circulation following intravenous injection of the standard formulation at an equivalent dose.

All pharmacokinetic analyses were conducted in accordance with ICH-GCP analytical guidelines to ensure data reliability and regulatory compliance. All animal experiments were designed and reported in accordance with the ARRIVE guidelines 2.0 to ensure methodological rigor and transparency.

### 2.9. Pharmacodynamic (PD) studies

The pharmacodynamic studies employed a grouping strategy outlined in Section 2.7. Rats received daily administrations of either a single 10 nmol/kg dose of the semaglutide standard formulation (described in Section 2.1) or saline (for the high-fat diet (HFD) and normal diet (ND) control groups) for 15 days. For sustained-release formulations, a single dose of 50 nmol/kg was given in every 5 days for 3 applications. During this period, the daily activity, body weight, and food intake of the rats were recorded. At the end of the experiment, the rats were fasted overnight, and their body weight, waistline, and body length were measured. Lee’s index was then calculated using the collected data (see Equation 3).

#### 2.9.1. Serum biochemical indicator tests

At the end of the pharmacodynamic studies, 0.8–1.0 mL of blood was collected from the jugular vein of rats. The blood samples were allowed to clot at room temperature for 2 h before centrifugation at 4 °C at 3000 rpm for 15 min. All assays were performed following the instructions provided with the Nanjing Jiancheng Bioengineering Institute reagent kits. Serum samples were stored at −80 °C. Serum triglyceride (TG) levels were measured using the CPO-PAP method: Lipoprotein lipase hydrolyzed TG to glycerol, which was subsequently converted via glycerol kinase and glycerol-3-phosphate oxidase to generate hydrogen peroxide (H_2_O_2_). The peroxide reacted with 4-AAP under peroxidase catalysis to form a red quinone derivative (*λ* = 500 nm), with quantification based on absorbance changes. Alanine aminotransferase (ALT) activity was determined by the Reitman-Frankel method: ALT catalyzed the transamination of L-alanine and α-ketoglutarate to produce pyruvate. After 30 min, 2,4-dinitrophenylhydrazine was added to terminate the reaction and form pyruvate phenylhydrazone, which developed a red-brown chromogen under alkaline conditions (*λ* = 510 nm). All assays were performed following the instructions provided with the Nanjing Jiancheng Bioengineering Institute reagent kits.

#### 2.9.2. Glucose tolerance tests

The glucose tolerance test (GTT) evaluates the body’s ability to regulate blood glucose levels, reflecting glucose metabolism and insulin resistance in rats. Following an overnight fast, a 50% glucose solution (BL1218A, Beijing Lanjieke Technology Co., Ltd.) was administered to three rats from each group at a dose of 2.0 g/kg. Blood glucose levels were measured using a blood glucose meter (MK1236, Jiangsu Yuyue Medical Equipment Co., Ltd.) before administration and at 15, 30, 60, and 120 min post-gavage. The resulting blood glucose levels were then plotted against time to generate a glucose tolerance curve for intergroup comparison.

#### 2.9.3. Tissue hematoxylin-eosin (HE) staining

At the end of the pharmacodynamic studies, three rats from each group were euthanized using excessive anesthesia with isoflurane (22082801, Rivard Life Technology Co., Ltd.). The liver, perirenal, and epididymal adipose tissues were quickly excised, washed with phosphate-buffered saline (PBS), dried with filter paper, and weighed. A portion of each liver tissue was fixed in 4% paraformaldehyde solution (BL539A, Beijing Lanjieke Technology Co., Ltd.) for histopathological examination via hematoxylin and eosin (HE) staining. The body fat index was calculated based on the collected data (see Equation 4).

(4) $$\text{BFI}=\frac{\text{Fat}\;\text{weight}(g)}{\text{Body}\;\text{weight}(g)}\times 100\%$$

### 2.10. Lifestyle experiments

During the drug treatment period, rats were randomly divided into two groups (Table 5) and subcutaneously injected with POE-SRP 50/100 in every 5 days with a duration of 15 days. After the drug treatment, rats were re-randomized into 6 groups, each with specific restrictions on diet type, daily food quantity, or feeding time. Throughout all the experiment, rats had unrestricted access to water. Their activity, food intake, and body weight were observed and recorded daily.

**Table 5.** Lifestyle experiment protocols.

During drug treatment		Randomized after 15 days of treatment →	Post drug treatment		
Group	Type of diet		Group	Type of diet	Dietary control measures
ND	Normal diet		NDHFD		Normal diet High-fat diet 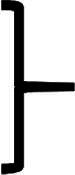	No restrictions on food quantity and feeding time
		
			ND 20 g*HFD 20 g	Normal diet High-fat diet 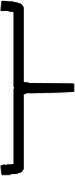	Daily dietary intake limited to 20 g, no restrictions on feeding time	
HFD	High-fat diet					
			ND 6 hHFD 6 h	Normal diet High-fat diet 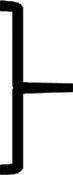	Feeding time limited to 6 h daily, no restrictions on daily dietary intake	
		

* Based on the average daily food intake of rats with same sizes.

### 2.11. Statistical analysis

All data are presented as mean ± standard deviation (*SD*). Data processing and statistical analysis were performed using SPSS 26.0 software. Results were considered statistically significant at *p* < 0.05 (*) and *p* < 0.01 (**). The results were visualized using GraphPad Prism 10. Pharmacokinetic parameters were estimated using Phoenix software (version 8.1, Pharsight) based on a non-compartmental model (NCA).

## 3. Results and discussion

### 3.1. Safety of the formulations and delivery methods

During the experiments, all rats in the normal diet (ND) group exhibited a healthy mental state, agile movement, and smooth, glossy fur. In contrast, rats in the high-fat diet (HFD) group displayed poor mental status, dry and yellowish fur, and sluggish movement. Following treatment with standard formulation or sustained-release formulation, the mental activity and coat condition of the HFD rats improved significantly. Throughout the entire study, no signs of toxicity, unexpected behavioral changes were observed in any of the rats, indicating good biocompatibility and safety of the sustained-release formulations and the micro-needle jet injector. Preliminary histological examination indicated that there is no damage to the skin epidermis, and there is no hard nodules and foreign body granuloma reaction in the dermis tissue.

### 3.2. Delivery efficiency of devices

DIO rats with shaved and hairy skin were used to evaluate the *in vivo* delivery efficiency of the extravascular delivery devices. The results are plotted in Figure 2(b). Traditional NI achieved 100% delivery efficiency in both rats with shaved and hairy skin. The NFJI produced results with large variations in rats with shaved skin, with delivery efficiency ranging from 40 to 80%. Frequent liquid leakages were observed at the delivery site; if the nozzle of the NFJI device was not firmly pressed on the skin, the incidence of liquid leakage would be greatly increased. In rats with unshaved hairy skin, the drug delivery efficiency of NFJI decreased sharply, ranging from 35 to 60%. The above-mentioned findings were consistent with the inconsistence of NFJI delivery that was reported in literatures (Rey et al. 2013; Cho et al. 2022). In contrast, MNJI, leveraging the skin penetration and targeted delivery benefits of NI, achieved 100% delivery efficiency in all rats, regardless of whether their skin was shaved or hairy. Thus, MNJI minimized skin and hair interference, provided accurate targeting, and eliminated the inconsistence and unpredictability associated with NFJI devices.

### 3.3. Initial dispersion rate of different delivery technologies

Figure 2(a) illustrates the initial dispersion rate of 5 different extravascular delivery devices, measured by a nanosponge-gel model. Previous studies have indicated that NFJI would increase drug dispersion at delivery leading to improvements in drug efficacy (Mao et al. 2023). As expected, the NFJI device in this study achieved a 50% higher initial dispersion rate than the traditional NI. The MNJI device, which employs a comparable jet mechanism of the NFJI device, achieved a dispersion rate comparable to NFJI under the equivalent operational parameters (nozzle size, driving force, etc.). By increasing micro-needle inner diameter from 133 to 232 μm, a 30% higher initial dispersion rate was achieved (sMNJI-1 *vs.* sMNJI-2). Increasing the number of micro-needles from one to three (sMNJI-1 *vs.* tMNJI) yielded an additional 56% increase in IDR (Figure 2(a)).

### 3.4. Pharmacokinetic study of the standard formulation

Table 6 shows the pharmacokinetic results of the semaglutide standard formulation delivered with traditional needle injection intravascularly (IV) and subcutaneously. The IV data was served as the baseline for the calculation of bioavailability. The half-life (*t*_1/2_) remained unchanged, suggesting that the metabolic mechanisms of semaglutide were unaffected by the route of administration. However, transitioning from intravascular to extravascular delivery, time to maximum concentration *T*_max_ significantly delayed, along with a flatting of the concentration curve and a significant decrease of the maximum drug concentration *C*_max_. Presumably, these delays are likely attributable to increased semaglutide metabolism before entering the bloodstream, as reflected in the area under the curve (AUC). These findings are consistent with previous reports (Lee et al. 2023). When the bioavailability of intravascular injection was set to 100%, the bioavailability of subcutaneous needle injection was calculated to be 42%.

**Table 6.** Pharmacokinetic parameters of standard formulation (single administration, *n* = 3, *M* ± *SD*).

PK parameters	*t*_1/2_ (h)	*t*_max_ (h)	*C*_max_ (ng·mL^−1^)	AUC_0–_*_t_* (h·ng·mL^−1^)	Bioavailability (%)
NI (IV)	9.08 ± 0.06	1.00 ± 0.00	1125.48 ± 58.58	12,274.41 ± 1456.54	100.00
NI	8.11 ± 2.05	6.00 ± 0.00	258.24 ± 68.74	5157.16 ± 798.47	42.02

In a separate experiment, pharmacokinetic studies were conducted with 5 different delivery devices. Figure 2(e) shows the pharmacokinetic concentration curves for these devices following a single dose of 10 nmol/kg semaglutide standard formulation. PK parameters were calculated and shown in Table 7. The bioavailability was calculated using AUC_0–_*_t_* relative to the AUC_0–_*_t_* of needle injection intravascularly.

**Table 7.** Pharmacokinetic parameters of standard formulation (single administration, *n* = 6, *M* ± *SD*).

Parameters	*t*_1/2_ (h)	*t*_max_ (h)	*C*_max_ (ng·mL^−1^)	AUC_0–_*_t_* (h·ng·mL^−1^)	Bioavailability (%)
NI	13.71 ± 1.99	6.00 ± 0.00	153.41 ± 70.25	4706.99 ± 1518.20	42.02 ± 13.55
NFJI	14.23 ± 0.26	10.00 ± 0.00	183.12 ± 26.86	5136.70 ± 1355.03	45.85 ± 12.10
sMNJI-1	13.77 ± 1.73	10.00 ± 0.00	173.04 ± 61.19	5146.51 ± 1443.27	45.94 ± 12.88
sMNJI-2	12.96 ± 1.85	10.00 ± 0.00	231.96 ± 78.43*	5790.66 ± 1130.78*	51.69 ± 10.09*
tMNJI	13.05 ± 1.18	10.00 ± 0.00	237.46 ± 91.04*	6304.82 ± 2006.22*	56.28 ± 17.91*

sMNJI-1: single micro-needle jet injection with small injection nozzle; sMNJI-2: single micro-needle jet injection with large injection nozzle; tMNJI: three-sites micro-needle jet injection.

*Note:* Bioavailability using NI group as baseline; *p < 0.10.

In all cases, the *t*_1/2_ remained consistent, indicating that the drug metabolism and the pharmacokinetic pathways remained unchanged despite the different initial dispersions from the different devices. Figure 2(c) shows the correlations between IDR and plasma concentrations at 30-min, 60-min, and *C*_max_. Figure 2(d) shows the correlation between IDR and bioavailability of semaglutide delivered by the 5 different devices. Linear relationships were observed in all cases, highlighting a direct dependence of plasma concentration and bioavailability on the initial dispersion rate. As expected, *T*_max_ increased along with the increase in bioavailability.

### 3.5. Pharmacodynamics of the standard formulation

#### 3.5.1. Body weight change

Before the pharmacodynamics studies, the average weight of the rats in the high-fat diet group was significantly higher than that of the normal group by 20%, indicating the successful establishment of the DIO rat model (Table 8). Following the drug treatment, the body weight in all treated rats was significantly lower than that in the HFD control group (*p* < 0.05). Likewise, waistline was significantly reduced (*p* < 0.05), and both BFI and Lee’s index were also reduced to some extent. Body weight in the treatment groups decreased rapidly after the initial administration and stabilized by the 6th day (see Figure 2(f)). Further injections of the same dose (10 nmol/kg) did not result in additional weight reduction from day 6 to day 15. Following cessation of injections at the end of the experiment, rapid weight recoveries occurred in all experimental rats. By day 25, most of the weight lost during drug treatment period was regained. Although there was no statistically significant difference in body weight changes among the NI, NFJI, and various MNJI groups, we did observe that NFJI group had a slightly less weight loss compared to the NI and MNJI groups. This outcome is likely attributable to the liquid leakage during NFJI injections (Figure 1(c)), as the higher dispersion rate of NFJI failed to compensate for the drug loss during administration. The higher weight loss ratio of sMNJI was consistent with the higher IDR of these devices. Interestingly, the tMNJI group showed a lower weight loss ratio, presumably due to the minor leakage observed from these lab prototype devices. Additional work is underway in our lab to improve these sophisticated prototype devices.

**Table 8.** Physical condition of rats (multiple administrations, *n* = 6, *M* ± *SD*).

Group	Body weight before treatment (g)	Body weight after treatment (g)	Body weight change (%)	Waistline (cm)	BFI (%)	Lee’s index
ND control	292.14 ± 7.73^##^	281.43 ± 12.05^##^	−3.94 ± 2.77^##^	14.38 ± 0.88^##^	0.44 ± 0.08^##^	279.69 ± 15.82^#^
HFD control	574.13 ± 52.57	576.00 ± 51.03	0.35 ± 1.05	20.38 ± 0.44	5.74 ± 1.26	304.72 ± 10.58
NI	571.13 ± 55.47	492.75 ± 54.77^##^	−12.53 ± 0.95^##^	19.13 ± 1.36^#^	4.87 ± 0.69	295.87 ± 3.83
NFJI	566.25 ± 60.46	499.13 ± 56.17^#^	−11.88 ± 2.42^##^	19.00 ± 0.96^##^	5.02 ± 1.53	302.76 ± 7.67
sMNJI-1	572.63 ± 53.06	497.25 ± 54.06^##^	−13.25 ± 3.11^##^	19.81 ± 0.84	4.03 ± 0.66	293.88 ± 5.60
sMNJI-2	577.88 ± 57.17	500.38 ± 50.98^##^	−13.42 ± 1.73^##^	19.19 ± 0.80^##^	4.27 ± 0.53	285.86 ± 8.14^#^
tMNJI	559.63 ± 44.56	495.25 ± 37.32^##^	−11.46 ± 2.14^##^	18.94 ± 0.86^##^	4.99 ± 1.40	286.30 ± 15.74

sMNJI-1: single micro-needle jet injection with small injection nozzle; sMNJI-2: single micro-needle jet injection with large injection nozzle; tMNJI: three-sites micro-needle jet injection.

*Note:* Compared with HFD group, ^#^*p* < 0.05, ^##^*p* < 0.01.

Overall, the observation that higher initial dispersion rates and bioavailability did not translate into statistically significant differences in weight change warrants further investigation. The authors suggest that in this study the GLP-1 plasma concentrations for all delivery methods were over-saturated, therefore masked the differences in efficacy enhancement of obesity management.

#### 3.5.2. Blood biochemical test

Obese individuals usually have impaired pancreatic function and abnormal glucose metabolism, placing them at risk for hyperlipidemia and fatty liver. Serum triglyceride (TG) and aniline aminotransferase (ALT) levels are important indicators of hyperlipidemia. As shown in Figure 2(g), the TG content and ALT activity of all groups of rats were similar, suggesting that the degree of obesity in the DIO rats did not reach the threshold for hyperlipidemia. Consequently, there were no significant differences in TG and ALT levels before or after treatment (*p* > 0.05).

#### 3.5.3. Glucose tolerance test

In the clinical studies reported by Friedrichsen et al, semaglutide has been shown to reduce body weight and blood glucose levels in overweight or obese people (with or without diabetes) (Blundell et al. 2017; Friedrichsen et al. 2021). As shown in Figure 2(h), the fasting blood glucose levels of rats treated with medication decreased to levels comparable to the ND control group. The results confirmed that semaglutide, as a glucose dependent GLP-1RA, could effectively reduce blood glucose and lower the risk of hypoglycemia. Following glucose gavage, the blood glucose levels of rats rapidly increased, but the blood glucose levels of all drug treatment groups were significantly lower than those in the HFD control group (*p* < 0.05), indicating that semaglutide effectively improved the abnormal glucose metabolism of rats and enhanced their ability to regulate blood glucose. No statistically significant differences in glucose tolerances were observed in the drug treated groups, regardless of the delivery method used for the macromolecular drug.

#### 3.5.4. Hematoxylin-eosin staining of semaglutide standard formulation

The HE staining images of liver tissue revealed notable differences among the experimental groups. The HE staining images (see Figure 2(i)) of liver tissue from the ND group showed well-preserved hepatic lobules. In the HFD control group, extensive fatty degeneration of hepatocytes was seen in the liver tissue, with round vacuoles of different sizes in the cytoplasm, and accumulation of lipid droplets. In the drug treatment groups, lipid droplets were significantly reduced. The treatment effect of the sMNJI-2 group, which exhibited the highest weight loss ratio, suggesting superior therapeutic efficacy compared to other delivery technologies.

In this study, although the higher IDR leads to a higher bioavailability with statistical significance, the pharmacodynamics results did not reveal statistically significant correlations between the IDR and the various clinical efficacy. This lack of correlation may be attributed to the dosing regimen used in this study. It is likely that the rats received an overdose of semaglutide, resulting in drug saturation at these dosages. This hypothesis will be further examined in our planned dose-ranging experiments. Consequently, higher bioavailability did not translate into enhanced efficacy. Additionally, the mechanism of GLP-1 action, as described in the introduction (Lau et al. 2015; Knudsen and Lau 2019; Gallo et al. 2022; Chuong et al. 2023), involves multiple pathways beyond pharmacokinetics, such as glucose regulation, appetite suppression, and gastrointestinal motility modulation. Thus, the impact of delivery methods on pharmacodynamics extends beyond the effects on initial dispersion and bioavailability. Future studies will focus on lower dosage regimens to explore these relationships further. Additional research, including clinical studies, is also planned to elucidate the interplay between delivery methods, pharmacokinetics, and the multifaceted mechanisms of GLP-1.

### 3.6. Delivery efficiency for sustained-release formulations

**Figure 3.**
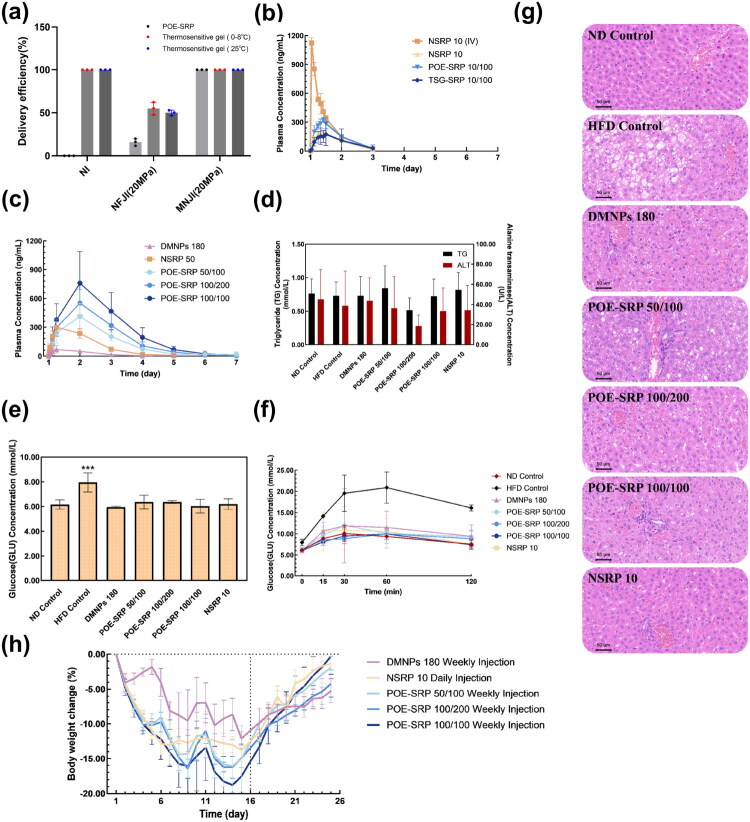
PK and PD results of sustained-release formulations. (a) Delivery efficiency of POE and TSG sustained-release formulations. The injection parameters for needle-free jet injection (NFJI) are: nozzle inner diameter of 150 μm and injection pressure of 20 MPa. The injection parameters for micro-needle jet injection (MNJI) are needle inner diameter of 232 μm, injection pressure of 14 MPa, and micro-needle length of 6 mm. (b) Plasma concentration curve of sustained-formulations in Table 9. (c) Plasma concentration curve of each group in Table 10. (d) Blood biochemical data (*n* = 8). (e) Fasting blood glucose after overnight fasting (*n* = 3). (f) Blood glucose changes in glucose tolerance test (*n* = 3). (g) HE staining of liver tissue (*n* = 3). The H&E staining images in Figures 2(j) and (g) represented the results of the standard formulation experiment and the sustained-release experiment, respectively. These experiments were conducted at the same time using the same batch of animals. In accordance with the animal study 3R principle (Reduction, Replacement, Refinement) to minimize the use of experimental animals, a shared control group was used for both experiments. (h) PD results of sustained-release formulations.

As shown in Figure 3(a), NI, NFJI, and MNJI exhibited markedly different delivery efficiencies for the sustained-release formulations in shaved skin. NI failed to deliver the highly viscous (∼1809.1 mPa·s) POE formulations into the subcutaneous layer, presumably due to lack of sufficient piston driving force. NFJI, although equipped with high piston driving force, experienced a significant leakage due to the skin barrier, leading to an injection efficiency of only 20%. In contrast, MNJI successfully overcame the injection challenges posed by these highly viscous formulations, achieving 100% delivery efficiency for all the POE sustained-release formulations.

Due to its unique thermal properties, the thermal sensitive gel sustained-release formulation exists in a sol state at 0–8 °C (∼65.53 mPa·s) and transitions to a gel state at ∼25 °C (∼3138 mPa·s). The TSG formulation was stored in a prefilled ampoule at low temperature to maintain its sol state and injected at the low temperature to achieve acceptable injection efficiency. As a non-Newtonians fluid, the thermal sensitive gel can be sheared during the injection. Both NI and MNJI successfully achieved 100% delivery efficiency, while NFJI had a significant leakage due to the skin barrier, leading to an injection efficiency of <50% (see Figure 3(a)). As noticed, TSG type formulations with different transition temperatures posted various challenges for NI and NFJI devices. However, because MNJI combines the skin penetration and targeted delivery feature of NI with the high piston driving force of NFJI, it achieved 100% delivery efficiency in all cases. With further optimizations, MNJI has the potential to become an alternative, but noninvasive, method to the traditional surgical operation for semi-solid or solid type sustained-release formulations.

### 3.7. Pharmacokinetic study of sustained-release formulations

#### 3.7.1. Pharmacokinetic parameters

Since bioavailability of macromolecular drugs could be dose dependent (Tomlinson et al. 2016), for sustained-release formulations, the bioavailability studies were conducted at the standard 10 nmol/kg dose level and the 50–100 nmol/kg dose levels. The pharmacokinetics results are shown in Tables 8 and 9, respectively.

**Table 9.** Pharmacokinetic parameters of formulations at 10 nmol/kg dosage (*n* = 3, *M* ± *SD*).

Parameters	*t*_1/2_ (h)	*t*_max_ (h)	*C*_max_ (ng·mL^−1^)	AUC_0–_*_t_* (h·ng·mL^−1^)	Bioavailability (%)
NI-NSRP 10 (IV)	9.08 ± 0.06	1.00 ± 0.00	1125.48 ± 58.58	12,274.41 ± 1456.54	100
NI-NSRP 10 (SC)	8.11 ± 2.05	6.00 ± 0.00	258.24 ± 68.74	5157.16 ± 798.47	42.02
MNJI-NSRP 10 (SC)	9.73 ± 0.00	9.00 ± 1.41	213.27 ± 22.92	5302.37 ± 453.69	43.19
MNJI-POE-SRP 10/100 (SC)	9.12 ± 0.00	10.00 ± 2.83	289.45 ± 52.07	8057.28 ± 2049.18	65.64
MNJI-TSG-SRP 10/100 (SC)	7.21 ± 0.00	11.00 ± 1.41	259.99 ± 57.40	8272.76 ± 1962.59	67.40

Table 9 shows the pharmacokinetic results of different formulations with drug load of 10 nmol/kg. The findings reveal that neither formulation nor delivery method alters the half-life of semaglutide. However, the time to reach peak plasma concentration was extended, from 1 h in the intravascular injection to 6 h in the needle injection of standard formulation, and further to 11 h in the MNJI of the sustained-released formulations. For extravascular injections, the *C*_max_ was consistent among groups and significantly lower than that observed in the intravascular injection group. Bioavailability in the MNJI group was slightly higher than the NI group, presumably due to the slight increase in initial dispersion rate. Bioavailability of both sustained-release formulations was substantially higher than that of the non-sustained-release formulations. This enhancement is attributed to the drug reservoir formed subcutaneously by the sustained-release formulations, which gradually release the drug by dissolution or diffusion through the depot surface, increasing the likelihood of absorption into the bloodstream (Adepu and Ramakrishna 2021).

Table 10 presents the pharmacokinetic results of different semaglutide formulations at 50–100 nmol/kg dose levels, using MNJI as the delivery method for consistency. The results reveal that, at 50–100 nmol/kg drug load, all POE sustained-release formulations achieved a similar half-life of ∼15 h. The 50–100 nmol/kg sustained-release formulation groups showed statistically longer half-life than that observed in the 10 nmol/kg standard formulation group. This may be attributed to the increase in the amount of drug in the subcutaneous reservoir which increase the amount of drug released per unit time as the delivery carrier dissolves. This slower release process may hinder drug entry into systemic circulation, resulting in a slight extension of the half-life.

**Table 10.** Pharmacokinetic parameters of formulations at 50–100 nmol/kg dosages (single administration, *n* = 6, *M* ± *SD*).

Parameters	*t*_1/2_ (h)	*t*_max_ (h)	*C*_max_ (ng·mL^−1^)	AUC_0–_*_t_* (h·ng·mL^−1^)	*V_z_*/*F* (mL·kg^−1^)	CL/F (mL·h^−1^·kg^−1^)	MRT (h)	Relative bioavailability (%)
NSRP 50	11.15 ± 1.29	6.00 ± 0.00	299.89 ± 59.09	10,998.69 ± 2308.05	343.03 ± 77.38	19.07 ± 3.90	23.71 ± 2.02	42.02
POE-SRP 50/100	13.99 ± 3.48	24.00 ± 0.00	411.57 ± 138.86	19,761.43 ± 6125.90	283.58 ± 129.39	11.07 ± 3.53	37.14 ± 8.35	75.49
POE-SRP 100/200	14.47 ± 1.65	24.00 ± 0.00	552.96 ± 141.33	31,596.33 ± 3088.80	340.81 ± 127.26	13.18 ± 1.56	36.78 ± 2.38	60.35
POE-SRP 100/100	15.55 ± 4.17	24.00 ± 0.00	759.16 ± 330.02	38,657.73 ± 16,163.60	279.59 ± 141.68	12.38 ± 5.27	39.34 ± 4.30	73.84
DMNPs	16.08 ± 8.14	6 ± 0.00	57.45 ± 38.37	1924.09 ± 1310.04	8381.13 ± 6755.57	375.93 ± 284.62	20.86 ± 5.32	2.04

*Note:* Non-sustained-release formulation (NSRP 50) at 42% in the previous study (Table 4) was used as baseline for calculation of relative bioavailability of sustained-release formulation. NSRP stands for Non-sustained-release preparations, referring to non-sustained release formulations (ordinary injections), where ‘10’ indicates a dose of 10 nmol/kg/day. POE-SRP stands for Poly(ortho ester)-Sustained-release preparations, referring to poly(ortho ester)-based sustained-release formulations, where ‘50’ indicates a single dose of 50 nmol/kg and ‘100’ indicates a single administration volume of 100 μL.

The time to reach peak plasma concentration had statistically significant extensions, increasing from 6 h in the non-sustained-release formulation group (NSRP 50) to 24 h in the non-sustained-release formulation group (POE-SRP). The subcutaneous drug reservoir formed by the sustained-release formulations facilitates gradual drug release through surface erosion, allowing for sustained drug entry into systemic circulation, which contributes to the increased *T*_max_. The *C*_max_ in the sustained-release formulation POE-SRP group was 1.5–2.5 times higher than that of the standard formulation NSRP 50 group. These findings clearly demonstrate that both sustained-release carriers substantially extended the bioavailability, further confirming the PD results shown in Figure 3(h).

Figures 3(b,c) present the time-concentration curve for semaglutide formulations at 10 nmol/kg dose and 50–100 nmol/kg dose, respectively. Both figures revealed the extension of *T*_max_ for the sustained-release formulations, from 6 h in the standard formulation group, to 12 h in the sustained-release formulations at 10 nmol/kg dose, then to 24 h in the sustained-release formulations at 50–100 nmol/kg doses. The extension of *T*_max_ is concentration dependent, suggesting that POE polymer delivery carriers can achieve sustained release of semaglutide through surface dissolution processes (Einmahl et al. 2000). In addition, the time concentration curve was prolonged to day 6. Its plasma concentration at day 4 was comparable to the *C*_max_ of the non-sustained release sample (NSRP 10) at 10 nmol/kg dose. This indicates that the POE polymer delivery carrier can extend the residence time of semaglutide to at least 4 days for DIO rats. Comparing sustained-release and non-sustained release samples at 50 nmol/kg dose, the plasma concentration of sustained-release sample on day 4 is comparable to the non-sustained release sample on day 2, further confirming the sustained-release capability of the POE carrier.

#### 3.7.2. Bioavailability

Table 8 also shows the relative bioavailability of the semaglutide with different delivery methods. At 10 nmol/kg dose, both POE and TSG carriers have substantially extended the bioavailability, from 43% in the MNJI-NSRP 10 to 65.64% in POE SRP group and 61.98% in TSG SRP group. In the 50 nmol/kg dose scenario, we calculated the relative bioavailability using the non-sustained-release at 42% as the baseline, both POE samples showed significant increase of bioavailability to above 60%, emphasizing the controlled-release and dissolution-release properties of the POE carrier.

The comparison of pharmacokinetic results from the POE-SRP 50/100 and POE-SRP 100/100 formulations are of interesting. When injection volume was maintained at the same level of 100 µL while semaglutide loading doubled from 50 to 100 nmol/kg, the *C*_max_ and AUC_0–_*_t_* nearly doubled. The relative bioavailability of these two formulations is similar and at high level of ∼75%. This indicates that the surface dissolution process of the polymer delivery carrier is highly stable, with the dissolution rate dependent on the carrier volume rather than the drug dosage. Conversely, comparing the POE-SRP 100/200 group with the POE-SRP 100/100 group (where semaglutide loading was maintained at the100 nmol/kg level but the injection volume increased from 100 to 200 µL), despite identical drug dosages, the larger carrier volume in the POE-SRP 100/200 group led to a reduction in both *C*_max_ and AUC_0–_*_t_*, as well as a dropping of relative bioavailability from 73.8 to 60.4%. This suggests that the increase in polymer delivery carrier volume will affect the surface dissolution rate, subsequently influencing drug absorption, plasma concentration, and the residence time of semaglutide.

Surprisingly, despite the significantly higher drug dose of DMNPs (180 nmol/kg), the experimental results were suboptimal. Visible watermarks were observed on the surface of the used patches, potentially due to the formation of a sealed environment on the skin. Prolonged exposure to such conditions may induce water secretion from the pores, potentially causing drug degradation and reducing therapeutic efficacy in the DMNPs group (Singh et al. 2024).

### 3.8. Pharmacodynamics of sustained-release formulations

#### 3.8.1. Blood biochemical test

The blood biochemistry data are shown in Figure 3(d), with comparison to the ND and HFD control groups. No significant differences (*p* > 0.05) were observed in serum triglyceride (TG) content or alanine aminotransferase (ALT) activity among the control and drug treatment groups.

#### 3.8.2. Glucose tolerance test

Fasting Glucose (GLU) levels are strong indicators of obesity induced metabolic syndrome (Alberti et al. 2009). As shown in Figure 3(e), the fasting GLU content in the HFD group was significantly higher than that of the ND control group and the drug treatment groups, whereas the fasting GLU level in the drug treatment group was comparable to that of the ND control group, with no statistically significant difference. After glucose gavage, the glucose recovery trend in the treatment group resembled that of the ND control group, and was substantially faster than that in the HFD control group (see Figure 3(f)). All rats treated with semaglutide showed a significant decrease in GLU content within 30 min after glucose gavage compared to the HFD control group (*p* < 0.05), with levels similar to those in the ND control group. These findings indicate that semaglutide effectively alleviates obesity-associated hyperglycemia while avoiding hypoglycemia due to excessively low glucose levels. Overall, semaglutide helps stabilize blood glucose within the physiological range and exerts a positive effect on glucose metabolism.

#### 3.8.3. Hematoxylin-eosin staining of sustained-release formulations

The Hematoxylin-Eosin (HE) staining images of liver tissue are shown in Figure 3(g). The ND control group exhibited intact hepatic lobule structure with no notable abnormalities. In the HFD control group, large number of fatty degeneration of hepatocytes were seen in the liver tissue, with round vacuoles of different sizes in the cytoplasm and accumulation of lipid droplets. The number and size of lipid droplets in HFD control group were significantly greater than the ND control group. After treatment with semaglutide, the number and size of lipid droplets were significantly reduced, approaching levels comparable to those observed in the ND control group.

#### 3.8.4. Body weight loss

The physical conditions of DIO rats and the treatment protocols are shown in Table 11. Before the experiment, the average body weight of rats in the high-fat diet group was 20% higher than that of the normal diet group, indicating the successful establishment of the DIO rat model. After the experiment, there was no significant change in body weight in the HFD group rats (0.35 ± 1.05%). All rats treated with medication showed a significant decrease in body weight compared to the HFD control group (*p* < 0.01), along with a certain degree of reduction in Waistline, BFI, and Lee’s index. The therapeutic effect of sustained-release formulation POE-SRP (3 injections in total 15 days) on rats reached the expected sustained-release effect, with the weight reduction significantly greater than that observed in the non-sustained-release formulation NSRP 10 group (15 daily injections). DMNPs group showed the smallest weight change after administration, with a decrease of only 7.29 ± 1.83%, which was significantly different from other drug treatment groups (*p* < 0.05).

**Table 11.** Physical condition of rats (multiple administrations, *n* = 8, *M* ± *SD*).

Group	Body weight before treatment (g)	Body weight after treatment (g)	Body weight change (%)	Waistline (cm)	BFI (%)	Lee’s index
ND control	292.14 ± 7.73**	281.43 ± 12.05**	−3.94 ± 2.77**	14.38 ± 0.88**	0.44 ± 0.08**	279.69 ± 15.82**
HFD control	574.13 ± 52.57	576.00 ± 51.03	0.35 ± 1.05	20.38 ± 0.44	5.74 ± 1.26	304.72 ± 10.58
DMNPs	597.83 ± 71.11	553.83 ± 61.56	−7.29 ± 1.83**	19.20 ± 0.45**	4.03 ± 0.77	291.08 ± 9.21
POE-SRP 50/100	582.14 ± 48.91	496.50 ± 44.05**	−14.66 ± 4.79**	20.14 ± 0.48	3.99 ± 1.70	289.17 ± 8.74
POE-SRP 100/200	589.00 ± 53.64	503.50 ± 49.49**	−14.56 ± 1.35**	19.19 ± 1.25*	4.94 ± 0.96	289.53 ± 10.47
POE-SRP 100/100	557.50 ± 48.04	462.38 ± 54.89**	−17.17 ± 4.63**	18.88 ± 1.06**	4.51 ± 1.52	290.93 ± 25.11
NSRP 10	577.88 ± 57.17	500.38 ± 50.98**	−13.42 ± 1.73**	19.19 ± 0.80**	4.27 ± 0.53	285.86 ± 8.14*

*Note:* Compared with HFD group, *p < 0.05, **p < 0.01.

Rats in the NSRP 10 group exhibited a plateau in weight loss after an initial weight reduction, indicating the emergence of an equilibrium phase. This plateau likely arises from prolonged semaglutide injections, during which the drug’s effect on gastric emptying diminishes over time, resulting in stabilized body weight. As shown in Figure 3(h), sustained release formulation POE-SRP overcame the limit of the equilibrium period leading to continued weight loss over 15 consecutive days.

The body weight changes of the POE-SRP formulation groups and the pharmacokinetic parameters shown good consistency. When injection volume was maintained at the same level of 100 µL while semaglutide loading doubled from 50 to 100 nmol/kg, the reduced body weight was significant, along with doubling of the *C*_max_ and AUC_0–_*_t_* as discussed in Section 3.8.1, supporting the dose-dependent therapeutic effect of semaglutide, where higher drug doses yielded more pronounced effects (Agarwal and Rupenthal 2013). Conversely, when comparing the POE-SRP 100/200 group with the POE-SRP 100/100 group that semaglutide loading was maintained at the 100 nmol/kg level while the injection volume increased from 100 to 200 µL, despite identical drug dosages, the larger carrier volume in the POE-SRP 100/200 group led to a reduction in both *C*_max_ and AUC_0–_*_t_*. as well as the relative bioavailability, in consequence, lowered body weight loss. In addition, Lee’s indexes of POE-SRP 100/100 group were significantly higher than those of POE-SRP 100/200 group (*p* > 0.05). Both indicate that the surface dissolution process of the POE polymer delivery carrier is very stable, with the dissolution rate dependent on the carrier volume rather than the drug dosage. Additionally, clinical applications of bupivacaine-loaded POE systems for postoperative or therapeutic pain management, providing long-lasting pain relief for ∼4–5 days, have been reported (Barr et al. 2002). This demonstrates the potential for similar sustained-release GLP-1 formulations to offer prolonged therapeutic effects for weight management.

In the cases of POE sustain-release formulations delivered by micro-needle jet injectors, study results suggest a clear relationship between pharmacokinetic parameters and pharmacodynamic outcomes. Thus, increased plasma concentration of semaglutide enhances its efficacy and promotes weight loss in DIO rats. Moreover, although current studies have demonstrated some efficacy in weight reduction, future research should focus on the composition of weight loss and promote healthy weight management (Karakasis et al. 2025).

### 3.9. Impact of diet control on weight management during drug treatment

**Figure 4.**
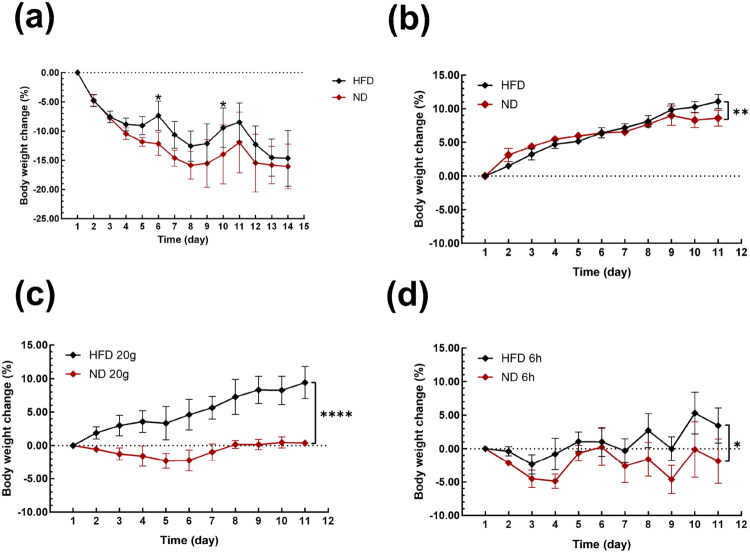
Diet control during and post drug treatment (a) during drug treatment. (b) Post drug treatment. No restrictions on food quantity and feeding time. (c) Daily dietary supply limited to 20 g. No restrictions on feeding time. (d) Feeding time limited to 6 h daily. No restrictions on daily dietary supply.

Semaglutide promotes weight loss primarily by reducing energy intake through appetite suppression and its neuroprotective effects (Svendstrup et al. 2024). As shown in Figure 4(a), diet control had a significant effect on the efficacy of semaglutide drug therapy. During the drug treatment, the final weight change in the ND group (16.07 ± 3.81%) was significantly lower than that in the HFD group (14.65 ± 4.79%), and the difference was statistically significant (*p* < 0.05). These results demonstrate that combining diet control with drug therapy can significantly improve weight-loss efficacy during the drug treatment.

### 3.10. Impact of diet control on weight management post drug treatment

Consistent with clinical observations in humans where rapid weight gain occurs after semaglutide discontinuation (Wilding et al. 2022), the experimental rats in this study also experienced rapid weight regain following the cessation of injections (as reported in Section 3.5.1). Within 10 days, most of the weight lost during drug treatment period was regained in all experimental rats.

In this study, we employed three diet control methods to study the impact of diet control on weight management post drug treatment. Among the three dietary strategies (see Figures 4(b–d)), the weight change of rats fed a high-fat diet was higher than that of rats fed a low-fat diet (*p* ≤ 0.05). There was no significant difference in food intake and body weight between the HFD group and the HFD 20g group (*p* > 0.05), indicating that the effect of high-fat diet on body weight is much greater than that of various dietary restriction strategies. However, simply switching from a high-fat diet to a normal diet, without additional controls, did not prevent fast weight gain. In both high-fat diet and normal diet groups, rats subjected to 6-h feeding restrictions exhibited significant weight fluctuations. This instability likely results from their nocturnal foraging behavior, as providing food during the day disrupts their natural feeding patterns, leading to irregular intake and weight changes. Regardless of whether they were on a high-fat or normal diet, the groups with a 6-h eating period demonstrated marked weight control. Almost all rat in these two groups maintained the weight loss during the 15 days. With on limitation on eating time, the group limited to 20 g of ND food supply effectively maintained weight loss (+0.36 ± 0.48%) after drug withdrawal, while the group limited to 20g of HFD food supply regained most of the weight loss from the treatment.

## 4. Conclusion

GLP-1 has emerged as an effective macromolecular drugs for weight control. While most current research focuses on prolonging the half-life of GLP-1 through chemical modification, this study approached obesity management from three perspectives: improving drug dispersion and bioavailability using high-speed jet injection, extending half-life with sustained-release formulations, and maintaining drug-induced weight loss through dietary control.

New micro-needle jet injectors (MNJI) with benefits of both needle injector (NI) in skin penetration and needle-free jet injectors (NFJI) in enhanced dispersion were developed (Long et al. 2025). The MNJI achieved 100% subcutaneous delivery efficiency for both standard and highly viscous sustained-release formulations, whereas NFJI frequently failed to penetrate the skin, and NI was unable to handle viscous formulations effectively.

High speed jet injection is capable of creating dispersion at the delivery site, potentially enhancing drug bioavailability. However, bioavailability enhancement from NFJI was inconsistent and unpredictable, likely due to incomplete skin penetration. In contrast, MNJI offers predictable delivery results. By adjusting device design parameters, a spectrum of dispersion patterns, quantified using the Initial Dispersion Rate (IDR), was achieved. For semaglutide, linear correlations between IDR and pharmacokinetic parameters, as well as relative bioavailability were revealed. As a result, MNJI increased the bioavailability of semaglutide from 42% (with NI) to 65% for standard formulation, and up to 75% for sustained-release formulations.

The non-sustained release formulations initially induced rapid weight loss in rats in the first 5 days, achieving comparable reductions in weight, blood glucose levels, and hepatic lipid droplets across all the delivery methods, despite different bioavailability. The weight loss reached a plateau after 5 continuous injections. This plateau in efficacy suggests a saturation effect at the 10 nmol/kg dose, masking potential differences in drug exposure. Further investigation is required to clarify this observation.

MNJI overcame the challenge of delivering highly viscous sustained-release formulations. Both POE and TSG formulations were successfully administered with 100% efficiency, leading to improved bioavailability as well *T*_max_ and *C*_max_. These enhancement in bioavailability had impact on weight management efficacy in two ways: (1) a single injection of the sustained-release formulation in every five days achieved same weight loss as five consecutive daily administrations of the standard formulation. (2) a second injection of led to further weight reduction, in sharp contrast to the plateau effect observed in the standard formulation with daily injections.

Diet control has significant impact on obesity management. During the drug treatment, rats fed a normal diet lost more weight than those on a high-fat diet, with statistically significant differences. However, weight rebound following treatment cessation was substantial in all groups without dietary intervention some animals even experiencing a ‘revenging rebound,’ exceeding their baseline weight. However, distinct differences emerged under specific diet control procedures. In the groups with a limited food supply but with no restriction on feeding-time, the high fat diet group exhibited a linear and rapid weight regain, while the normal diet group largely sustained its weight loss at −18%. Conversely, in the groups with a feeding time limited to 6 h daily but with unrestricted food supply, both ND and HFD groups effectively maintained their weight loss at the −18% level.

In summary, MNJI-enabled delivery of sustained release GLP-1 formulations significantly improved drug bioavailability and weight management outcomes. The technology enabled efficient, noninvasive administration of highly viscous formulations, extended dosing intervals from daily to once every five days, and facilitated better patient compliance. Combined with structured dietary protocols (especially time-restricted feeding) this drug-device-diet strategy presents a promising long-term therapeutic approach for clinical obesity management.

## Data Availability

The data supporting the findings of this study are available from the corresponding author upon reasonable request.
